# Psychological and Physical Stress Response and Incidence of Irregular Menstruation in Female University Employees: A Retrospective Cohort Study

**DOI:** 10.2188/jea.JE20240424

**Published:** 2025-10-05

**Authors:** Yuichiro Matsumura, Ryohei Yamamoto, Maki Shinzawa, Yuko Nakamura, Sho Takeda, Masayuki Mizui, Isao Matsui, Yusuke Sakaguchi, Asami Yagi, Yutaka Ueda, Chisaki Ishibashi, Kaori Nakanishi, Daisuke Kanayama, Hiroyoshi Adachi, Izumi Nagatomo

**Affiliations:** 1 Laboratory of Behavioral Health Promotion, Department of Health Promotion Medicine, Graduate School of Medicine, The University of Osaka, Osaka, Japan; 2 Health and Counseling Center, The University of Osaka, Osaka, Japan; 3 Department of Nephrology, Graduate School of Medicine, The University of Osaka University, Osaka, Japan; 4 Institute for Sports and Global Health, The University of Osaka, Osaka, Japan; 5 Department of Obstetrics and Gynecology, Graduate School of Medicine, The University of Osaka, Osaka, Japan

**Keywords:** occupational stress, psychological stress, irregular menstruation, menstrual cycle irregularity, menstrual disorders

## Abstract

**Background:**

This study aimed to assess the clinical relevance of three-dimensional occupational stress (job stressor score [A score], psychological and physical stress response score [B score], and social support for workers score [C score]) of the Brief Job Stress Questionnaire (BJSQ) in the national stress check program in Japan to irregular menstruation.

**Methods:**

The present retrospective cohort study included 2,078 female employees aged 19–45 years who had both annual health checkups and the BJSQ between April 2019 and March 2022 in a national university in Japan. The outcome was self-reported irregular menstruation measured at annual health checkups until March 2023. A dose-dependent association between BJSQ scores and incidence of irregular menstruation was examined using Cox proportional hazards models to calculate multivariable-adjusted hazard ratios (HRs) of four quantile (0–49% [Q_0–49_], 50–74% [Q_50–74_], 75–89% [Q_75–89_], and 90–100% [Q_90–100_]) of the BJSQ scores.

**Results:**

During 2.0 years of the median observational period, 257 (12.4%) women reported irregular menstruation. B score, not A or C scores, was identified as a significant predictor of irregular menstruation (adjusted HR of A, B, and C scores per 1 standard deviation: 1.06 [95% confidence interval {CI}, 0.89–1.27], 1.35 [95% CI, 1.15–1.57], and 0.93 [95% CI, 0.80–1.08], respectively). Women with higher B score had a significantly higher risk of irregular menstruation in a dose-dependent manner (adjusted HR of Q_0–49_, Q_50–74_, Q_75–89_, and Q_90–100_: 1.00 [reference], 1.38 [95% CI, 1.00–1.90], 1.48 [95% CI, 1.00–2.18], and 2.18 [95% CI, 1.38–3.43], respectively).

**Conclusion:**

Psychological and physical stress response predicted irregular menstruation.

## INTRODUCTION

The menstrual cycle is controlled by the interaction between hormones produced by the ovaries, hypothalamus, and pituitary glands.^1^ A normal menstrual cycle indicates normal endocrine and productive health, but 14–25% women experience menstrual cycle irregularity,^2^ which is commonly defined as >35 days or <21 days,^2^ and feel negative effect on quality of life (QOL).^3^^,^^4^ Moreover, previous studies suggested that irregular menstruation was associated with various health problems, including tinnitus,^5^ obesity,^6^ type 2 diabetes mellitus,^7^ metabolic syndrome,^8^ coronary heart disease,^9^^,^^10^ and ovarian cancer.^11^ Treatment of irregular menstruation can lead to improvements in women’s QOL and health.^2^

Occupational stress is a pivotal factor for many health problems, including depression,^12^ suicide,^13^ infertility,^14^ menopause,^15^ breast cancer,^16^ obesity,^17^ cardiovascular disease,^18^ and stroke.^19^ Regarding irregular menstruation, some cross-sectional studies reported conflicting association of occupational stress with irregular menstruation. A Taiwanese cross-sectional study with 746 female nurses reported an association between occupational stress and irregular menstruation,^20^ whereas an Iranian cross-sectional study with 150 midwives did not.^21^ Although the National Institute of Occupational Safety and Health of the Centers for Disease Control and Prevention classified occupational stress into a three-dimensional composites of job stressors, psychological and physical stress response, and individual and social factors,^22^ few studies have not compared their clinical impacts on irregular menstruation among these dimensions of occupational stress. Regarding menopause, some cross-sectional studies reported that low support^23^ and low job control^24^ were associated with menopause. The association between these dimensions of occupational stress and irregular menstruation should be assessed in cohort studies.

The aim of this retrospective cohort study was to examine the association between occupational stress and the incidence of self-reported irregular menstruation in 2,078 female employees at a national university. The Brief Job Stress Questionnaire (BJSQ) was used to measure occupational stress, which has been adopted by the national stress check program since December 2015 in Japan.^25^^,^^26^ This study elucidated that psychological and physical stress response, one of three dimensions of the BJSQ, predicted irregular menstruation, whereas other two dimensions did not.

## METHODS

### Study population

**Figure 1.**
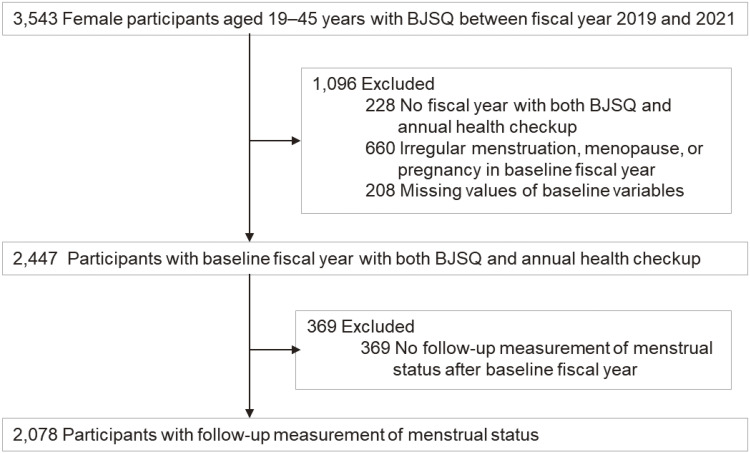
Flow diagram of inclusion and exclusion of study participants. BJSQ, brief job stress questionnaire.

Eligible participants of this retrospective cohort study included 3,543 female employees of the University of Osaka aged 19–45 years who completed the BJSQ in the national stress check program during the entry period between fiscal years 2019 and 2021 (April 2019 and March 2022) (Figure 1). A response rate of the BJSQ in the University of Osaka was 62.0%, 69.2%, 72.0%, and 66.8% in fiscal year 2019, 2020, 2021, and 2022, respectively. We excluded 228 (6.4%) women who did not have both the BJSQ and annual health checkups in a single fiscal year during the entry period; 660 (18.6%) with irregular menstruation, menopause, or pregnancy in the baseline fiscal year; and 208 (5.9%) with missing values of baseline variables. The baseline fiscal year was set in the first fiscal year with both the BJSQ and annual health checkup during the entry period. We set the baseline date on the date when women answered the BJSQ in the baseline fiscal year. The median days from the date of the BJSQ to that of the annual health checkups was 21 (interquartile range: 6–39). After excluding 369 (10.4%) women with no follow-up measurement of self-reported menstrual status during the 4-year study period until the end of March 2023, the present study included 2,078 (58.7%) women finally.

### Ethical considerations

The Ethics Committee of the Health and Counseling Centre, the University of Osaka approved the study protocol (No. 2024-1). According to the Japanese Ethical Guidelines for Medical and Health Research Involving Human Subjects, we used an opt-out approach to obtain informed consent of the participants. All procedures were conducted in accordance with the ethical standards of our institution and with 1964 Declaration of Helsinki and its later amendments.

### Measurements

The main exposure of interest was the baseline BJSQ scores of three dimensions, which were based on (i) 17 questionnaires about job stressors (A score) (ii) 29 questionnaires about psychological and physical stress response (B score), including the six response aspects of vigor, anger irritability, fatigue, anxiety, depressive mood, and physical responses, and (iii) 9 questionnaires about social support for workers (C score).^27^ All questionnaires were measured on a four-point Likert-type scale (1 = almost never, 2 = sometimes, 3 = often, and 4 = almost always). A, B, and C scores potentially ranged between 17 (= 1 × 17) and 68 (= 4 × 16), 29 (= 1 × 29) and 116 (= 4 × 29), and 9 (= 1 × 9) and 36 (= 4 × 9), respectively. High scores indicated a high degree of job stressors and stress responses, and a low degree of social support.

Baseline variables included age (years), body mass index (body weight [kg] ÷ height^2^ [m^2^]), systolic and diastolic blood pressures (mm Hg), drinking frequency, smoking status, breakfast frequency, weekly exercise frequency, night shift frequency, posture at work, TV watching time, hemoglobin A1c (%), and low-density lipoprotein cholesterol (mg/dL) at the baseline health checkups in the baseline fiscal year. Drinking frequency was measured according to the questionnaire “How often do you drink alcohol per week?” with possible responses of “0 day,” “1–2 days,” “3–4 days,” “5–6 days,” and “every day.” Regarding smoking history, the following questionnaire was asked: “Have you ever smoked?” with three possible responses of “I have never smoked,” “I quit smoking,” and “I am smoking now.” Breakfast frequency was asked by the questionnaire “How many times did you have breakfast in a week?” with possible responses of “0 day,” “1–2 days,” “3–4 days,” “5–6 days,” and “every day.” Regarding weekly exercise frequency, “How many times do you exercise in a week?” was asked with possible answers of “0 day,” “1 day,” “2 days,” “3–4 days,” and “≥5 days.” Night shift frequency was ascertained using the question “How many days a month do you work late at night between 22:00 pm to 5:00 am?” with possible answers of “0 time” “1–2 times,” “3–5 times,” “6–10 times,” and “≥11 times.” Exercise frequency was based on the question “How many times do you exercise in a week?” with possible responses of “0 day,” “1 day,” “2 days,” “3–4 days,” and “≥5 days.” Working posture was asked with five possible options of “sitting,” “standing,” “walking,” “carrying,” and “heavy labor” for which multiple selections were allowed. TV watching time was assessed using the question “How many hours do you watch TV in a day?” with possible answers of “0–10 minutes,” “10–30 minutes,” “30–60 minutes,” “1–2 hours,” and “≥2 hours.”

The outcome measure of interest was self-reported irregular menstruation, which was based on the questionnaire; “Is your current menstrual cycle regular?” The four possible answers were “regular,” “irregular,” “menopausal,” and “pregnant.” The observational period was defined as time from the date of the baseline BJSQ to (i) the date of the incident outcome, or (ii) the date of the last measurement of menstrual status at annual health checkups until the end of March 2023, whichever came first.

### Statistical analysis

To compare the baseline characteristics of included women with those of excluded women, the chi-squared test and the student’s *t*-test were used as appropriate. To compare those of women with different BJSQ scores, women were classified into four quantile categories of the BJSQ scores: 0–49% (Q_0–49_), 50–74% (Q_50–74_), 75–89% (Q_75–89_), and 90–100% (Q_90–100_). The baseline characteristics were compared among women with the four quantile categories of the BJSQ scores using the chi-squared test or analysis of variance.

The cumulative probability of the incident irregular menstruation was estimated using the Kaplan–Meier method. The cumulative probabilities of women with Q_50–74_, Q_75–89_, and Q_90–100_ of the BJSQ scores were compared with those with Q_0–49_, using the log-rank test. The association between the baseline BJSQ scores and the incident irregular menstruation was assessed using unadjusted and multivariable-adjusted Cox-proportional hazards models for calculation of hazard ratios (HRs) with 95% confidence intervals (CIs). Covariates in multivariable-adjusted models included baseline fiscal year (2019, 2020, and 2021), age (years), sex, body mass index (kg/m^2^), systolic blood pressure (mm Hg), drinking frequency (0, 1–2, 3–4, 5–6, and 7 days/week), smoking status (never, past, and current smokers), breakfast frequency (0, 1–2, 3–4, 5–6, and 7 days/week), weekly exercise frequency (0, 1, 2, 3–4, and ≥5 days/week), night shift frequency (0, 1–2, 3–5, 6–10, and ≥11 times/month), posture at work (sitting, standing, walking, carrying, physical work), TV watching time (0–10, 10–30, 30–60 minutes, 1–2 hours, and ≥2 hours/day), hemoglobin A1c (%), low-density lipoprotein cholesterol level (mg/dL), A score (if B and C scores), B score (if A and C scores), and C score (if B and C scores). The Schoenfeld residuals were used to check the proportional hazard assumption.

To exclude perimenopausal irregular menstruation, subgroup analysis stratified by age <40 and ≥40 years was also conducted, because menopause is rare in women under 40 years of age.^28^

Categorical variables were expressed as numbers (proportions) and continuous variables as median (25–75%) or mean (standard deviation), as appropriate. *P*-value of <0.05 was regarded as being statistically significant. We used Stata version 16.1 (Stata Corp, College Station, TX, USA) for all statistical analyses.

## RESULTS

After excluding 228 (6.4%) women with no fiscal year with both health checkup and the BJSQ and 660 (18.6%) women with irregular menstruation, menopause, or pregnancy at the baseline health checkup, the baseline clinical characteristics of 2,078 (58.7%) women included in the present study were compared with those of 577 (16.3%) women excluded from the present study, who had missing values of baseline variables (*n* = 208 [5.9%]), and no follow-up measurement of menstrual status after the baseline fiscal year (*n* = 369 [10.4%]) (Figure 1). Included women were slightly older than exclude women (mean age 34.8; standard deviation [SD], 7.1 vs 31.5; SD, 7.1 years). However, other baseline variables were clinically comparable between included women and excluded women, despite their statistical differences because of a large sample size (eTable 1).

The baseline characteristics of women with four quantile categories of the A, B, and C scores are listed in eTables 1, Table 1, and eTable 2, respectively. Women with Q_90–100_ of A scores were younger and had a higher prevalence of current smokers than those with Q_0–49_ (eTable 2). Women with Q_90–100_ of B scores were younger, had a higher prevalence of current smokers, higher frequency of night shifts, and longer TV watching time than those with Q_0–49_ (Table 1). Women with Q_90–100_ of C scores were older and had a higher prevalence of current smokers than those with Q_0–49_ (eTable 3).

**Table 1.** Baseline characteristics of 2,078 female employees stratified by psychological and physical stress response score (B score) of brief job stress questionnaire (BJSQ)

		All	Quantiles of psychological and physical stress response score (range)			

			Q_0–49_ (29–51)	Q_50–74_ (52–61)	Q_75–89_ (62–71)	Q_90–100_ (72–113)
Number		2,078	1,004	543	315	216
Age, years		34.8 (7.1)	35.5 (7.0)	34.4 (7.1)	34.1 (7.3)	33.8 (7.2)
Body mass index, kg/m^2^		21.2 (3.0)	21.1 (2.7)	21.2 (3.2)	21.5 (3.1)	21.7 (3.7)
Systolic blood pressure, mm Hg		110.7 (12.5)	110.7 (12.0)	110.2 (13.1)	111.2 (12.8)	111.5 (13.0)
Diastolic blood pressure, mm Hg		66.0 (9.2)	65.9 (8.8)	65.8 (9.4)	66.0 (9.5)	67.0 (9.7)
Drinking frequency,	0 day/week	1,094 (52.6)	530 (52.8)	288 (53.0)	158 (50.2)	118 (54.6)
	1–2	687 (33.1)	330 (32.9)	177 (32.6)	112 (35.6)	68 (31.5)
	3–4	147 (7.1)	72 (7.2)	41 (7.6)	18 (5.7)	16 (7.4)
	5–6	67 (3.2)	31 (3.1)	19 (3.5)	9 (2.9)	8 (3.7)
	7	83 (4.0)	41 (4.1)	18 (3.3)	18 (5.7)	6 (2.8)
Smoking status,	Never	1,875 (90.2)	918 (91.4)	483 (89.0)	287 (91.1)	187 (86.6)
	Stop smoking	157 (7.6)	75 (7.5)	48 (8.8)	18 (5.7)	16 (7.4)
	Smoking	46 (2.2)	11 (1.1)	12 (2.2)	10 (3.2)	13 (6.0)
Breakfast frequency,	0 day/week	89 (4.3)	30 (3.0)	31 (5.7)	11 (3.5)	17 (7.9)
	1–2	176 (8.5)	63 (6.3)	46 (8.5)	39 (12.4)	28 (13.0)
	3–4	152 (7.3)	48 (4.8)	50 (9.2)	29 (9.2)	25 (11.6)
	5–6	231 (11.1)	86 (8.6)	63 (11.6)	48 (15.2)	34 (15.7)
	7	1,430 (68.8)	777 (77.4)	353 (65.0)	188 (59.7)	112 (51.9)
Weekly exercise,	0 day/week	1,035 (49.8)	466 (46.4)	264 (48.6)	179 (56.8)	126 (58.3)
	1	555 (26.7)	277 (27.6)	152 (28.0)	81 (25.7)	45 (20.8)
	2	267 (12.8)	133 (13.2)	80 (14.7)	32 (10.2)	22 (10.2)
	3–4	141 (6.8)	82 (8.2)	30 (5.5)	14 (4.4)	15 (6.9)
	≥5 days	80 (3.8)	46 (4.6)	17 (3.1)	9 (2.9)	8 (3.7)
Night working,	0 days/month	1,526 (73.4)	806 (80.3)	381 (70.2)	217 (68.9)	122 (56.5)
	1–2	131 (6.3)	58 (5.8)	36 (6.6)	26 (8.3)	11 (5.1)
	3–5	316 (15.2)	114 (11.4)	97 (17.9)	47 (14.9)	58 (26.9)
	6–10	87 (4.2)	19 (1.9)	25 (4.6)	22 (7.0)	21 (9.7)
	≥11 days	18 (0.9)	7 (0.7)	4 (0.7)	3 (1.0)	4 (1.9)
Posture at work,	Sitting	1,375 (66.2)	725 (72.2)	341 (62.8)	194 (61.6)	115 (53.2)
	Standing	466 (22.4)	192 (19.1)	137 (25.2)	82 (26.0)	55 (25.5)
	Walking	205 (9.9)	79 (7.9)	58 (10.7)	32 (10.2)	36 (16.7)
	Carrying	6 (0.3)	2 (0.2)	1 (0.2)	1 (0.3)	2 (0.9)
	Physical work	26 (1.3)	6 (0.6)	6 (1.1)	6 (1.9)	8 (3.7)
TV watching time,	0–10 min/day	286 (13.8)	129 (12.8)	79 (14.5)	41 (13.0)	37 (17.1)
	10–30	416 (20.0)	212 (21.1)	105 (19.3)	57 (18.1)	42 (19.4)
	30–60	709 (34.1)	364 (36.3)	172 (31.7)	125 (39.7)	48 (22.2)
	1–2 hour	514 (24.7)	237 (23.6)	142 (26.2)	71 (22.5)	64 (29.6)
	≥2 hours	153 (7.4)	62 (6.2)	45 (8.3)	21 (6.7)	25 (11.6)
Hemoglobin A1c, %		5.2 (0.3)	5.2 (0.2)	5.2 (0.3)	5.2 (0.3)	5.2 (0.3)
LDL cholesterol, mg/dL		109.5 (26.2)	108.7 (25.4)	109.1 (25.7)	111.5 (27.2)	111.3 (29.3)

LDL, low-density lipoprotein.

Number (%) or mean (standard deviation).

*P* < 0.05 for all variables among four quantile categories of B score, except drinking frequency.

**Figure 2.**
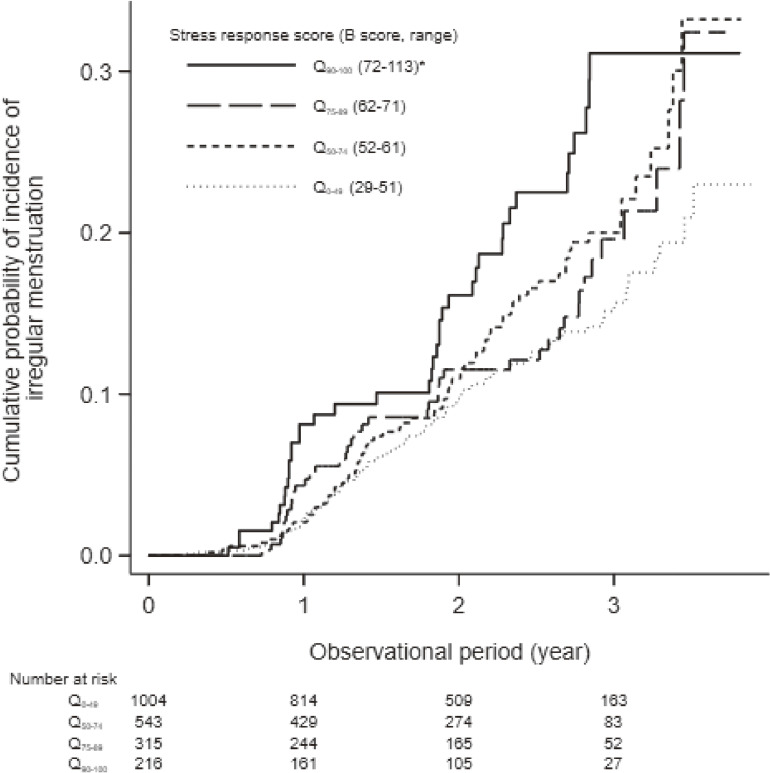
Psychological and physical stress response score (B score) and incidence of irregular menstruation in 2,078 female employees. ^*^P < 0.05 vs Q_0–49_

Irregular menstruation was reported by 257 (12.4%) women during the median observational period of 2.01 (interquartile range, 1.08–2.84) years. Women with Q_90–100_ of B scores had significantly higher cumulative probability of irregular menstruation than those with Q_0–49_ (Figure 2), whereas no significant difference was observed regarding A or C scores (eFigure 1 and eFigure 2). Multivariable-adjusted models identified B score as a significant predictor of irregular menstruation, whereas not A or C scores (adjusted HR per 1 standard deviation of A, B, and C scores: 1.06 [95% CI, 0.89–1.27], 1.35 [95% CI, 1.15–1.57], and 0.93 [95% CI, 0.80–1.08], respectively) (Table 2).

**Table 2.** Associations of job stressor score (A score), psychological and physical stress response score (B score) and social support score for workers (C score) with incidence of irregular menstruation

	Job stressor score (A score)	Psychological and physical stress response score (B score)	Social support score for workers (C score)
All, *n* = 2,078			
Unadjusted HR (95% CI), per 1 SD	1.07 (0.92–1.26)	1.25 (1.08–1.46)^*^	0.94 (0.82–1.09)
Adjusted HR (95% CI), per 1 SD^a^	1.06 (0.89–1.27)	1.35 (1.15–1.57)^*^	0.93 (0.80–1.08)

Age <40 years, *n* = 1,388			
Unadjusted HR (95% CI), per 1 SD	1.20 (0.97–1.48)	1.14 (0.93–1.40)	0.88 (0.72–1.08)
Adjusted HR (95% CI), per 1 SD^a^	1.03 (0.81–1.32)	1.25 (1.01–1.56)^*^	0.92 (0.75–1.13)

Age ≥40 years, *n* = 690			
Unadjusted HR (95% CI), per 1 SD	0.99 (0.78–1.25)	1.46 (1.18–1.82)^*^	0.94 (0.76–1.17)
Adjusted HR (95% CI), per 1 SD^a^	0.96 (0.75–1.24)	1.60 (1.27–2.01)^*^	0.95 (0.76–1.20)

CI, confidence interval; HR, hazard ratio; SD, standard deviation.

^*^P < 0.05.

^a^Adjusted for baseline fiscal year (2019, 2020, and 2021), age (years), sex, body mass index (kg/m^2^), systolic blood pressure (mm Hg), drinking frequency (0, 1–2, 3–4, 5–6, and 7 days/week), smoking status, breakfast frequency (0, 1–2, 3–4, 5–6, and 7 days/week), weekly exercise frequency (0, 1, 2, 3–4, and ≥5 days/week), night working (0, 1–2, 3–5, 6–10, and ≥11 days/month), posture at work (sitting, standing, walking, carrying, and physical work) and TV watching time (0–10 minutes, 10–30 minutes, 30–60 minutes, 1–2 hours, and ≥2 hours/day), hemoglobin A1c, low-density lipoprotein cholesterol (mg/dL), A score (if B and C scores), B score (if A and C scores), and C score (if A and B scores).

To clarify the dose-dependent association between B score and the incidence of irregular menstruation, we calculated unadjusted and multivariable-adjusted HRs of Q_0–49_, Q_50–74_, Q_75–89_, and Q_90–100_ of B score. The incident irregular menstruation was observed in 101 (10.1%), 72 (13.3%), 44 (14.0%), and 40 (18.5%) women with Q_0–49_, Q_50–74_, Q_75–89_, and Q_90–100_ of B score, respectively (Table 3 and Figure 2). The unadjusted model showed that women with Q_50–74_ and Q_90–100_ of B score had a significantly higher risk of irregular menstruation than those with Q_0–49_ scores (Table 3). Even after adjusting for covariates, the dose-dependent association between B score and irregular menstruation was evident (adjusted HR of Q_0–49_, Q_50–74_, Q_75–89_, and Q_90–100_: 1.00 [reference], 1.38 [95% CI, 1.00–1.90], 1.48 [95% CI, 1.00–2.18], and 2.18 [95% CI, 1.38–3.43], respectively) (Table 3). Regarding A and C scores, their four quantile categories were not associated with irregular menstruation in the multivariable-adjusted models (eTable 4 and eTable 5).

**Table 3.** Psychological and physical stress response score (B score) and incidence of irregular menstruation

	Quantile categories of psychological and physical stress response score (score range)			

	Q_0–49_ (29–51)	Q_50–74_ (52–61)	Q_75–89_ (62–71)	Q_90–100_ (72–113)
All, *n* = 2,078				
Number	1,004	543	315	216
Menopause, *n* (%)^a^	7 (0.7)	6 (1.1)	3 (1.0)	0 (0.0)
Irregular menstruation, *n* (%)	101 (10.1)	72 (13.3)	44 (14.0)	40 (18.5)
Observational period, year	1.99 (1.10, 2.85)	1.99 (1.08, 2.84)	2.06 (1.04, 2.86)	1.92 (0.97, 2.80)
IR per 1,000 PY (95% CI)	51.1 (42.1–62.2)	68.5 (54.3–86.3)	69.1 (51.5–92.9)	99.8 (73.2–136.1)
Unadjusted HR (95% CI)	1.00 (reference)	1.36 (1.00–1.84)^*^	1.33 (0.93–1.89)	2.02 (1.40–2.91)^*^
Adjusted HR (95% CI)^b^	1.00 (reference)	1.38 (1.00–1.90)^*^	1.48 (1.00–2.18)^*^	2.18 (1.38–3.43)^*^

Age <40 years, *n* = 1,388				
Number	633	375	221	159
Menopause, *n* (%)^a^	0 (0.0)	1 (0.3)	0 (0.0)	0 (0.0)
Irregular menstruation, *n* (%)	54 (8.5)	40 (10.7)	24 (10.9)	23 (14.5)
Observational period, year	1.96 (1.11, 2.85)	1.96 (1.12, 2.84)	2.02 (1.07, 2.84)	1.90 (0.98, 2.79)
IR per 1,000 PY (95% CI)	45.0 (34.4–58.7)	56.5 (41.5–77.1)	54.6 (36.6–81.5)	78.8 (52.4–118.6)
Unadjusted HR (95% CI)	1.00 (reference)	1.26 (0.84–1.89)	1.20 (0.74–1.94)	1.79 (1.10–2.91)^*^
Adjusted HR (95% CI)^b^	1.00 (reference)	1.16 (0.75–1.80)	1.29 (0.75–2.21)	1.96 (1.06–3.61)^*^

Age ≥40 years, *n* = 690				
Number	371	168	94	57
Menopause, *n* (%)^a^	7 (1.9)	5 (3.0)	3 (3.2)	0 (0.0)
Irregular menstruation, *n* (%)	47 (12.7)	32 (19.0)	20 (21.3)	17 (29.8)
Observational period, year	2.02 (1.09, 2.87)	2.05 (1.05, 2.80)	2.28 (0.98, 2.89)	2.03 (0.92, 2.82)
IR per 1,000 PY (95% CI)	60.7 (45.6–80.8)	93.0 (65.8–131.6)	101.6 (65.5–157.4)	156.1 (97.0–251.1)
Unadjusted HR (95% CI)	1.00 (reference)	1.57 (1.00–2.47)^*^	1.61 (0.95–2.72)	2.71 (1.55–4.73)^*^
Adjusted HR (95% CI)^b^	1.00 (reference)	1.66 (1.02–2.70)^*^	1.80 (1.00–3.23)^*^	3.35 (1.62–6.91)^*^

CI, confidence interval; HR, hazard ratio; IR, incidence rate; PY, person year.

Observational period was expressed as median (25%–75%).

^*^P < 0.05.

^a^Participants with menopause after incidence of irregular menstruation.

^b^Adjusted for baseline fiscal year (2019, 2020, and 2021), age (years), sex, body mass index (kg/m^2^), systolic blood pressure (mm Hg), drinking frequency (0, 1–2, 3–4, 5–6, and 7 days/week), smoking status, breakfast frequency (0, 1–2, 3–4, 5–6, and 7 days/week), weekly exercise frequency (0, 1, 2, 3–4, and ≥5 days/week), night working (0, 1–2, 3–5, 6–10, and ≥11 times/month), posture at work (sitting, standing, walking, carrying, and physical work), TV watching time (0–10 minutes, 10–30 minutes, 30–60 minutes, 1–2 hours/day, and ≥2 hours/day), HbA1c, LDL cholesterol, job stressor score (A score), and social support score for workers (C score).

To examine the influence of perimenopausal irregular menstruation, subgroup analyses stratified by <40 and ≥40 years of age were conducted. Among 1,388 women aged <40 years, including only one woman who reported menopause after the incidence of irregular menstruation, the B score was associated with irregular menstruation in a dose-dependent manner, similar to the findings in all women (Table 2 and Table 3), while the A and C scores were not (Table 2). Among the 690 women aged ≥40 years, 15 reported menopause after the incidence of irregular menstruation. A similar dose-dependent association was observed among women aged ≥40 years (Table 2 and Table 3), whereas the A and C scores were not (Table 2).

## DISCUSSION

This retrospective cohort study disclosed that female employees with higher psychological and physical stress response was at a higher risk of irregular menstruation than those with those with lower response. Some advantages of the present study were: first, its retrospective cohort study design; second, the large number of participants (*n* = 2,078), which allowed for a statistically meaningful assessment; and third, the direct comparison of clinical impacts on the incidence of irregular menstruation among three dimensions of occupational stress. These findings clarify that psychological and physical stress affect irregular menstruation, which may affect women’s QOL and presenteeism.^29^

The conflicting associations between occupational stress and irregular menstruation were reported in previous cross-sectional studies. A Chinese cross-sectional study, which included 12,881 nurses and health care workers aged 18–50 years,^30^ and a Taiwanese cross-sectional study, which included 746 female nurses aged 20–45 years, showed a significant association between occupational stress and irregular menstruation.^20^ In contrast, an Iranian cross-sectional study with 150 midwives aged 20–40 years showed no statistically significant differences.^21^ This retrospective cohort study, with a robust study design, a large number of participants, and a long observational period, confirmed the clinical impact of psychological and physical stress response of the BJSQ on irregular menstruation.

One of the plausible mechanisms linking occupational stress to the incidence of irregular menstruation may be dysregulation of the hypothalamic-pituitary-adrenal (HPA) axis. An American observational study with 285 police officers showed an association between occupational stress and decreased daytime cortisol secretion due to HPA dysregulation.^31^ HPA dysregulation can cause gonadotropin-releasing hormone disorder and irregular menstruation,^32^ suggesting that occupational stress potentially cause irregular menstruation. Further studies are required to clarify the mechanism linking occupational stress and irregular menstruation.

This study has some limitations. First, the participants of this study were employees of a single national university with a low response rate of the BJSQ, while some previous studies reported a high response rate of the BJSQ (94%^33^ and 85.7%^34^). The generalizability of the results should be verified using different cohorts with a high response rate of the BJSQ. Second, exclusion of 369 women with no follow-up measurement of menstrual status after the baseline fiscal year might lead to underestimation of the association between occupational stress and incident irregular menstruation, because occupational stress is a critical factor of occupational turnover.^33^ Women with high occupational stress might have quitted their job before incident irregular menstruation was measured at annual health checkups in the present study. Given that high occupational stress may promote occupational turnover, the present study may have underestimated the clinical impact of occupational stress on the incidence of irregular menstruation. Third, occupational stress measured using the BJSQ was underestimated by social desirability bias,^35^ in which the employees did not give the correct answer due to concern about personnel disadvantages. Objective stress markers, such as cortisol,^36^^,^^37^ may be useful to assess the clinical impact of mental stress and the incidence of irregular menstruation. Fourth, the length of irregular menstrual cycles was not measured in this study. A Korean cross-sectional study with 9,335 women aged 22–45 years reported that workload, defined as standing work for more than 9 hours, was associated with short cycle menstruation.^38^ The clinical impact of occupational stress on the length of the menstrual cycle should be clarified in future studies. Fifth, although the BJSQ aims to measure occupational stress,^26^ it was not clear whether psychological and physical stress response was occupational in the present study or not. Underlying medical condition leading potentially to irregular menstruation might affect 29 symptoms of psychological and physical stress response, including polycystic ovary syndrome,^39^ thyroid disorders,^40^ uterine or ovarian diseases,^41^ and breast cancer^42^; medications, such as contraceptives,^43^ steroids,^44^ and psychiatric drugs^45^; and treatments for cancers.^46^ Unfortunately, information on such medical condition was not available in the present study, which should be assessed in a future well-designed study.

In conclusion, this retrospective cohort study identified psychological and physical stress response of the BJSQ as significant predictor of irregular menstruation among 2,078 female university employees. These findings are clinically useful to identify women valuable to irregular menstruation. The clinical impact of psychological and physical stress response on irregular menstruation should be verified in well-designed cohort studies.

## SUPPLEMENTARY MATERIAL

eTable 1. Baseline characteristics of 2,078 included and 577 excluded female employees

eTable 2. Baseline characteristics of 2,078 female employees stratified by job stressor score (A score) of brief job stress questionnaire (BJSQ)

eTable 3. Baseline characteristics of 2,078 female employees stratified by social support for workers score (C score) of brief job stress questionnaire (BJSQ)

eTable 4. Job stressor score (A score) and incidence of irregular menstruation

eTable 5. Social support score for workers (C score) and incidence of irregular menstruation

eFigure 1. Job stressor score (A score) and incidence of irregular menstruation in 2,078 female employees. P > 0.05 for Q_50–74_, Q_75–89_, and Q_90–100_ vs. Q_0–49_

eFigure 2. Social support score for workers (C score) and incidence of irregular menstruation in 2,078 female employees. P > 0.05 for Q_50–74_, Q_75–89_, and Q_90–100_ vs. Q_0–49_
