# Supplementary material for: Psychological and Physical Stress Response and Incidence of Irregular Menstruation in Female University Employees: A Retrospective Cohort Study
Source: J Epidemiol. 2025 Oct 5;35(10):425–31. doi: 10.2188/jea.JE20240424 (PMC12420952; doi:10.2188/jea.JE20240424)
Supplement: Supplementary file 1 [file je-35-425-s001.pdf]

eTable 1. Baseline characteristics of 2,078 included and 577 excluded female employees

		Included	Excluded	Missing, n (%)
Number		2,078	577	
Age, years		34.8 (7.1)	31.5 (7.1)	0 (0.0)
Body mass index, kg/m^2		21.2 (3.0)	21 (3.0)	133 (23.1)
Systolic blood pressure, mm Hg		110.7 (12.5)	109.7 (12.0)	133 (23.1)
Diastolic blood pressure, mm Hg		66.0 (9.2)	65.4 (9.1)	133 (23.1)
Drinking frequency,	0 day/week	1,094 (52.6)	308 (53.8)	4 (0.7)
	1–2	687 (33.1)	191 (33.3)	
	3–4	147 (7.1)	47 (8.2)	
	5–6	67 (3.2)	13 (2.3)	
	7	83 (4.0)	14 (2.4)	
Smoking status,	Never	1,875 (90.2)	527 (91.3)	0 (0.0)
	Stop smoking	157 (7.6)	39 (6.8)	
	Smoking	46 (2.2)	8 (1.4)	
Breakfast frequency,	0 day/week	89 (4.3)	38 (6.6)	2 (0.3)
	1–2	176 (8.5)	48 (8.4)	
	3–4	152 (7.3)	41 (7.1)	
	5–6	231 (11.1)	65 (11.3)	
	7	1,430 (68.8)	383 (66.6)	
Weekly exercise,	0 day/week	1,035 (49.8)	284 (49.9)	8 (1.4)
	1	555 (26.7)	157 (27.6)	
	2	267 (12.8)	67 (11.8)	
	3–4	141 (6.8)	40 (7.0)	
	≥5 days	80 (3.8)	21 (3.7)	
Night working,	0 days/month	1,526 (73.4)	422 (75.1)	15 (2.6)
	1–2	131 (6.3)	51 (9.1)	
	3–5	316 (15.2)	60 (10.7)	
	6–10	87 (4.2)	20 (3.6)	
	≥11 days	18 (0.9)	9 (1.6)	
Posture at work,	Sitting	1,375 (66.2)	335 (59.2)	11 (1.9)
	Standing	466 (22.4)	157 (27.7)	
	Walking	205 (9.9)	64 (11.3)	
	Carrying	6 (0.3)	5 (0.9)	
	Physical work	26 (1.3)	3 (0.5)	
TV watching time,	0–10min/day	286 (13.8)	113 (19.7)	4 (0.7)
	10–30	416 (20.0)	134 (23.4)	

	30–60	709 (34.1)	158 (27.6)	
	1–2 hour	514 (24.7)	132 (23.0)	
	≥2 hours	153 (7.4)	36 (6.3)	
Hemoglobin A1c, %,		5.2 (0.3)	5.2 (0.4)	34 (5.9)
LDL cholesterol, mg/dL		109.5 (26.2)	107.5 (28.0)	34 (5.9)

LDL, low-density lipoprotein.

Number (%) and mean (standard deviation)

$P<0.05$ for all variables

eTable 2. Baseline characteristics of 2,078 female employees stratified by job stressor score (A score) of brief job stress questionnaire (BJSQ**)**

		All	Quartiles of job stressor score (range)			
			$Q_{0–49}$ (17–38)	$Q_{50–74}$ (39–43)	$Q_{75–89}$ (44–47)	$Q_{90–100}$ (48–68)
Number		2,078	978	529	304	267
Age, years		34.8 (7.1)	36.3 (6.6)	34.5 (7.0)	33.0 (7.5)	32.1 (7.5)
Body mass index, kg/m^2		21.2 (3.0)	21.2 (3.0)	21.3 (3.0)	21.4 (3.1)	21.3 (3.1)
Systolic blood pressure, mm Hg		110.7 (12.5)	110.7 (12.5)	111.5 (13.3)	110.0 (12.0)	109.8 (11.6)
Diastolic blood pressure, mm Hg		66.0 (9.2)	65.9 (9.0)	66.7 (9.6)	66.0 (9.0)	65.2 (9.1)
Drinking frequency,	0 day/week	1,094 (52.6)	539 (55.1)	275 (52.0)	143 (47.0)	137 (51.3)
	1–2	687 (33.1)	285 (29.1)	177 (33.5)	123 (40.5)	102 (38.2)
	3–4	147 (7.1)	80 (8.2)	36 (6.8)	14 (4.6)	17 (6.4)
	5–6	67 (3.2)	31 (3.2)	19 (3.6)	12 (3.9)	5 (1.9)
	7	83 (4.0)	43 (4.4)	22 (4.2)	12 (3.9)	6 (2.2)
Smoking status,	Never	1,875 (90.2)	881 (90.1)	476 (90.0)	278 (91.4)	240 (89.9)
	Stop smoking	157 (7.6)	83 (8.5)	39 (7.4)	18 (5.9)	17 (6.4)
	Smoking	46 (2.2)	14 (1.4)	14 (2.6)	8 (2.6)	10 (3.7)
Breakfast frequency,	0 day/week	89 (4.3)	33 (3.4)	19 (3.6)	15 (4.9)	22 (8.2)
	1–2	176 (8.5)	60 (6.1)	44 (8.3)	36 (11.8)	36 (13.5)
	3–4	152 (7.3)	54 (5.5)	45 (8.5)	26 (8.6)	27 (10.1)
	5–6	231 (11.1)	92 (9.4)	67 (12.7)	34 (11.2)	38 (14.2)
	7	1,430 (68.8)	739 (75.6)	354 (66.9)	193 (63.5)	144 (53.9)
Weekly exercise,	0 day/week	1,035 (49.8)	456 (46.6)	246 (46.5)	176 (57.9)	157 (58.8)
	1	555 (26.7)	267 (27.3)	153 (28.9)	75 (24.7)	60 (22.5)
	2	267 (12.8)	128 (13.1)	80 (15.1)	33 (10.9)	26 (9.7)
	3–4	141 (6.8)	80 (8.2)	33 (6.2)	11 (3.6)	17 (6.4)
	≥5 days	80 (3.8)	47 (4.8)	17 (3.2)	9 (3.0)	7 (2.6)
Night working,	0 days/month	1,526 (73.4)	883 (90.3)	361 (68.2)	167 (54.9)	115 (43.1)
	1–2	131 (6.3)	37 (3.8)	51 (9.6)	24 (7.9)	19 (7.1)
	3–5	316 (15.2)	46 (4.7)	93 (17.6)	83 (27.3)	94 (35.2)
	6–10	87 (4.2)	3 (0.3)	20 (3.8)	28 (9.2)	36 (13.5)
	≥11 days	18 (0.9)	9 (0.9)	4 (0.8)	2 (0.7)	3 (1.1)
Posture at work,	Sitting	1,375 (66.2)	838 (85.7)	334 (63.1)	128 (42.1)	75 (28.1)
	Standing	466 (22.4)	102 (10.4)	130 (24.6)	110 (36.2)	124 (46.4)
	Walking	205 (9.9)	33 (3.4)	61 (11.5)	61 (20.1)	50 (18.7)
	Carrying	6 (0.3)	1 (0.1)	0 (0.0)	3 (1.0)	2 (0.7)

	Physical work	26 (1.3)	4 (0.4)	4 (0.8)	2 (0.7)	16 (6.0)
TV watching time,	0–10min/day	286 (13.8)	144 (14.7)	71 (13.4)	36 (11.8)	35 (13.1)
	10–30	416 (20.0)	209 (21.4)	115 (21.7)	46 (15.1)	46 (17.2)
	30–60	709 (34.1)	337 (34.5)	181 (34.2)	110 (36.2)	81 (30.3)
	1–2 hour	514 (24.7)	218 (22.3)	129 (24.4)	84 (27.6)	83 (31.1)
	≥2 hours	153 (7.4)	70 (7.2)	33 (6.2)	28 (9.2)	22 (8.2)
Hemoglobin A1c, %,		5.2 (0.3)	5.2 (0.2)	5.2 (0.3)	5.2 (0.3)	5.2 (0.3)
LDL cholesterol, mg/dL		109.5 (26.2)	110.5 (26.2)	109.6 (26.7)	108.5 (25.7)	106.7 (25.7)

LDL, low-density lipoprotein.

Number (%) and mean (standard deviation)

$P<0.05$ for all variables among four quartile categories of A score, except smoking status and TV watching time

eTable 3. Baseline characteristics of 2,078 female employees stratified by social support for workers score (C score) of brief job stress questionnaire (BJSQ**)**

		All	Quartiles of job stressor score (range)			
			$Q_{0–49}$ (9–17)	$Q_{50–74}$ (18–21)	$Q_{75–89}$ (22–24)	$Q_{90–100}$ (25–36)
Number		2,078	880	655	302	241
Age, years		34.8 (7.1)	34.2 (7.3)	34.9 (7.1)	35.6 (6.8)	36.1 (6.6)
Body mass index, kg/m^2		21.2 (3.0)	21.2 (2.8)	21.0 (2.9)	21.8 (3.5)	21.5 (3.4)
Systolic blood pressure, mm Hg		110.7 (12.5)	110.7 (12.6)	110.1 (12.3)	111.2 (13.1)	111.8 (12.3)
Diastolic blood pressure, mm Hg		66.0 (9.2)	65.7 (9.1)	65.9 (9.1)	66.5 (9.5)	66.8 (9.4)
Drinking frequency,	0 day/week	1,094 (52.6)	445 (50.6)	352 (53.7)	164 (54.3)	133 (55.2)
	1–2	687 (33.1)	303 (34.4)	225 (34.4)	86 (28.5)	73 (30.3)
	3–4	147 (7.1)	71 (8.1)	36 (5.5)	22 (7.3)	18 (7.5)
	5–6	67 (3.2)	26 (3.0)	17 (2.6)	14 (4.6)	10 (4.1)
	7	83 (4.0)	35 (4.0)	25 (3.8)	16 (5.3)	7 (2.9)
Smoking status,	Never	1,875 (90.2)	799 (90.8)	605 (92.4)	262 (86.8)	209 (86.7)
	Stop smoking	157 (7.6)	66 (7.5)	41 (6.3)	29 (9.6)	21 (8.7)
	Smoking	46 (2.2)	15 (1.7)	9 (1.4)	11 (3.6)	11 (4.6)
Breakfast frequency,	0 day/week	89 (4.3)	31 (3.5)	19 (2.9)	21 (7.0)	18 (7.5)
	1–2	176 (8.5)	61 (6.9)	51 (7.8)	28 (9.3)	36 (14.9)
	3–4	152 (7.3)	50 (5.7)	59 (9.0)	18 (6.0)	25 (10.4)
	5–6	231 (11.1)	92 (10.5)	72 (11.0)	45 (14.9)	22 (9.1)
	7	1,430 (68.8)	646 (73.4)	454 (69.3)	190 (62.9)	140 (58.1)
Weekly exercise,	0 day/week	1,035 (49.8)	431 (49.0)	321 (49.0)	154 (51.0)	129 (53.5)
	1	555 (26.7)	219 (24.9)	184 (28.1)	87 (28.8)	65 (27.0)
	2	267 (12.8)	121 (13.8)	83 (12.7)	39 (12.9)	24 (10.0)
	3–4	141 (6.8)	64 (7.3)	48 (7.3)	17 (5.6)	12 (5.0)
	≥5 days	80 (3.8)	45 (5.1)	19 (2.9)	5 (1.7)	11 (4.6)
Night working,	0 days/month	1,526 (73.4)	676 (76.8)	477 (72.8)	211 (69.9)	162 (67.2)
	1–2	131 (6.3)	52 (5.9)	43 (6.6)	18 (6.0)	18 (7.5)
	3–5	316 (15.2)	127 (14.4)	105 (16.0)	40 (13.2)	44 (18.3)
	6–10	87 (4.2)	20 (2.3)	22 (3.4)	31 (10.3)	14 (5.8)
	≥11 days	18 (0.9)	5 (0.6)	8 (1.2)	2 (0.7)	3 (1.2)
Posture at work,	Sitting	1,375 (66.2)	588 (66.8)	434 (66.3)	194 (64.2)	159 (66.0)
	Standing	466 (22.4)	188 (21.4)	149 (22.7)	73 (24.2)	56 (23.2)
	Walking	205 (9.9)	91 (10.3)	62 (9.5)	32 (10.6)	20 (8.3)
	Carrying	6 (0.3)	2 (0.2)	2 (0.3)	0 (0.0)	2 (0.8)

	Physical work	26 (1.3)	11 (1.3)	8 (1.2)	3 (1.0)	4 (1.7)
TV watching time,	0–10min/day	286 (13.8)	125 (14.2)	78 (11.9)	48 (15.9)	35 (14.5)
	10–30	416 (20.0)	174 (19.8)	141 (21.5)	57 (18.9)	44 (18.3)
	30–60	709 (34.1)	323 (36.7)	223 (34.0)	81 (26.8)	82 (34.0)
	1–2 hour	514 (24.7)	199 (22.6)	163 (24.9)	84 (27.8)	68 (28.2)
	≥2 hours	153 (7.4)	59 (6.7)	50 (7.6)	32 (10.6)	12 (5.0)
Hemoglobin A1c, %		5.2 (0.3)	5.2 (0.3)	5.2 (0.2)	5.2 (0.3)	5.2 (0.3)
LDL cholesterol, mg/dL		109.5 (26.2)	108.4 (26.3)	109.4 (26.3)	112.8 (26.5)	109.6 (24.7)

LDL, low-density lipoprotein.

Number (%) or mean (standard deviation)

$P<0.05$ for all variables among four quartile categories of C score, except drinking frequency, breakfast frequency, weekly exercise, and posture at work.

eTable 4. Job stressor score (A score) and incidence of irregular menstruation

	Quartiles of job stressor score (score range)			
	$Q_{0–49}$ (17–38)	$Q_{50–74}$ (39–43)	$Q_{75–89}$ (44–47)	$Q_{90–100}$ (48–68)
N (Total n=2,078)	978	529	304	267
Menopause, n (%)[a]	11 (1.1)	4 (0.8)	0 (0.0)	1 (0.4)
Irregular menstruation, n (%)	111 (11.3)	56 (10.6)	43 (14.1)	47 (17.6)
Observational period, year	2.03 (1.15, 2.84)	1.98 (1.03, 2.86)	2.04 (1.10, 2.84)	1.93 (1.03, 2.79)
IR per 1,000 PY (95% CI)	57.0 (47.3–68.7)	54.6 (42.0–71.0)	71.6 (53.1–96.6)	95.6 (71.8–127.2)
Unadjusted HR (95% CI)	1.00 (reference)	0.98 (0.71–1.36)	1.31 (0.92–1.86)	1.81 (1.29–2.55)*
Adjusted HR (95% CI)[b]	1.00 (reference)	0.96 (0.61–1.52)	1.32 (0.70–2.47)	1.80 (0.80–4.04)

CI, confidence interval; HR, hazard ratio; IR, incidence rate; PY, person year.

Observational period was expressed as median (25%–75%).

*P<0.05

[a]Participants with menopause after incidence of irregular menstruation.

[b]Adjusted for baseline fiscal year (2019, 2020, and 2021), age (years), sex, body mass index (kg/m^2), systolic blood pressure (mm Hg), drinking frequency (0, 1–2, 3–4, 5–6, and 7 days/week), smoking status, breakfast frequency (0, 1–2, 3–4, 5–6, and 7 days/week), weekly exercise frequency (0, 1, 2, 3–4, and ≥5 days/week), night working (0, 1–2, 3–5, 6–10 , and ≥11 days/month), posture at work (sitting, standing, walking, carrying, and physical work), TV watching time (0–10 minutes, 10–30 minutes, 30–60 minutes, 1–2 hours/day, and ≥2 hours/day), hemoglobin A1c (%), low-density lipoprotein cholesterol (mg/dL), psychological and physical stress response score (B score), and social support score for workers (C score).

eTable 5. Social support score for workers (C score) and incidence of irregular menstruation

	Quartiles of social support score for workers (score range)			
	$Q_{0–49}$ (9–17)	$Q_{50–74}$ (18–21)	$Q_{75–89}$ (22–24)	$Q_{90–100}$ (25–36)
N (Total n=2,078)	880	655	302	241
Menopause, n (%)[a]	6 (0.7)	4 (0.6)	2 (0.7)	4 (1.7)
Irregular menstruation, n (%)	101 (11.5)	86 (13.1)	32 (10.6)	38 (15.8)
Observational period, year	2.01 (1.09, 2.84)	2.04 (1.16, 2.85)	2.01 (1.02, 2.84)	1.97 (0.97, 2.84)
IR per 1,000 PY (95% CI)	58.7 (48.3–71.3)	65.8 (53.3–81.3)	55.1 (39.0–77.9)	83.4 (60.7–114.6)
Unadjusted HR (95% CI)	1.00 (reference)	1.11 (0.83–1.47)	0.93 (0.63–1.39)	1.41 (0.97–2.05)
Adjusted HR (95% CI)[b]	1.00 (reference)	1.02 (0.64–1.63)	0.83 (0.40–1.71)	1.25 (0.50–3.08)

CI, confidence interval; HR, hazard ratio; IR, incidence rate; PY, person year.

Observational period was expressed as median (25%–75%).

*P<0.05

[a]Participants with menopause after incidence of irregular menstruation.

[b]Adjusted for baseline fiscal year (2019, 2020, and 2021), age (years), sex, body mass index (kg/m^2), systolic blood pressure (mm Hg), drinking frequency (0, 1–2, 3–4, 5–6, and 7 days/week), smoking status, breakfast frequency (0, 1–2, 3–4, 5–6, and 7 days/week) weekly exercise frequency (0, 1, 2, 3–4, and ≥5 days/week), night working (0, 1–2, 3–5, 6–10 , and ≥11 days/month), posture at work (sitting, standing, walking, carrying, and physical work), TV watching time (0–10 minutes, 10–30 minutes, 30–60 minutes, 1–2 hours/day, and ≥2 hours/day), hemoglobin A1c (%), low-density lipoprotein cholesterol (mg/dL), job stressor score (A score), and psychological and physical stress response score (B score).

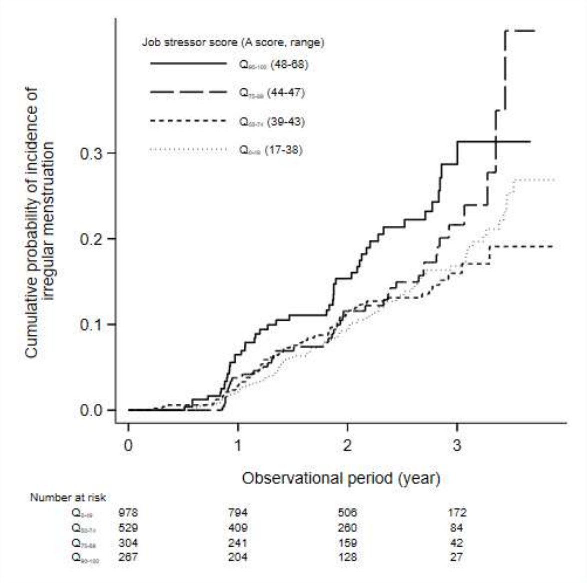

eFigure 1. Job stressor score (A score) and incidence of irregular menstruation in 2,078 female employees. P>0.05 for $Q_{50–74}$, $Q_{75–89}$, and $Q_{90–100}$ vs. $Q_{0–49}$

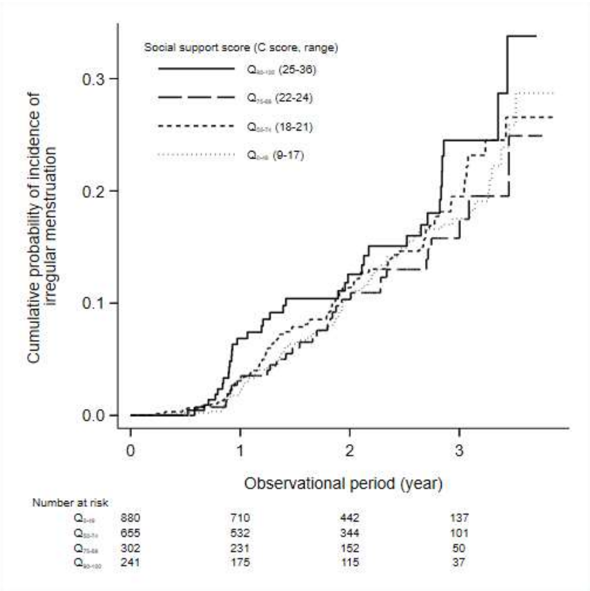

eFigure 2. Social support score for workers (C score) and incidence of irregular menstruation in 2,078 female employees. P>0.05 for $Q_{50–74}$, $Q_{75–89}$, and $Q_{90–100}$ vs. $Q_{0–49}$
